# Sperm Capacitation and Kinematics in *Phodopus* Hamsters

**DOI:** 10.3390/ijms242216093

**Published:** 2023-11-08

**Authors:** Ana Sanchez-Rodriguez, Ingrid I. D. Idrovo, Juan Antonio Rielo, Eduardo R. S. Roldan

**Affiliations:** Department of Biodiversity and Evolutionary Biology, Museo Nacional de Ciencias Naturales (CSIC), Calle Jose Gutierrez Abascal 2, 28006 Madrid, Spain; anasanchez@mncn.csic.es (A.S.-R.);

**Keywords:** capacitation, hyperactivation, sperm kinematics, motility, progesterone, hamster, *Phodopus*

## Abstract

This study was designed to analyze changes in the spermatozoa of three species of *Phodopus* hamsters incubated under different conditions. Cauda epididymal sperm were incubated for 4 h in modified Tyrode’s medium containing albumin, lactate, pyruvate, and Hepes (mTALP-H), in the same medium with the addition of bicarbonate (mTALP-BH), or with bicarbonate and 20 ng/mL of progesterone (mTALP-BH+P4). Media with bicarbonate are believed to promote capacitation in rodent species. Sperm motility, viability, capacitation patterns, and kinematics were assessed at different times. Capacitation in live cells was quantified after staining with Hoechst 33258 and chlortetracycline. Patterns believed to correspond to non-capacitated cells (F pattern), capacitated, acrosome-intact cells (B pattern), and acrosome-reacted cells (AR pattern) were recognized. Kinematics were examined via computer-assisted sperm analysis (CASA). The results showed a decrease in total motility in all three species in different media, with a sharp decrease in progressive motility in bicarbonate-containing media (without or with progesterone), suggesting hyperactivated motion. However, none of the other signs of hyperactivation described in rodents (i.e., decrease in STR or LIN, together with an increase in ALH) were observed. F pattern cells diminished with time in all media and were generally lower in *P. roborovskii* and higher in *P. campbelli*. B pattern cells increased in mTALP-BH media in all species. Progesterone did not enhance the percentage of B pattern cells. Finally, AR pattern cells increased in all species incubated in different media, showing the highest percentage in *P. roborovskii* and the lowest in *P. campbelli*. Comparisons between media revealed that there were higher percentages of F pattern cells and lower percentages of B pattern cells over time in medium without bicarbonate (mTALP-H) in comparison to media containing bicarbonate (mTALP-BH; mTALP-BH+P4). Overall, changes consistent with the acquisition of capacitation and development of hyperactivated motility were found; however, further studies are required to better characterize media necessary to support the pathways involved in these processes in *Phodopus* species.

## 1. Introduction

Sperm “capacitation” was originally defined as the set of processes that spermatozoa undergo in the female tract and that are needed for fertilization [1]. It was first uncovered in rats [2] and in rabbits [2,3] by showing that sperm cells did not enter the egg until several hours after the injection in the rat’s periovarian sac or into the rabbit’s oviduct. Many studies have been conducted to understand the process of capacitation. Currently, the term capacitation is generally used in a more restrictive way, as many authors reserve this term for the processes occurring before acrosomal exocytosis (the so-called “acrosome reaction”) [4,5,6,7]; the latter involves the fusion of the outer acrosomal membrane with the plasma membrane and is needed for the sperm to penetrate the oocyte [8]. Other authors still argue that capacitation includes all the events occurring in the female tract [9,10], which would, by definition, include acrosomal exocytosis. When spermatozoa undergo capacitation, they change their pattern of movement, loosing progressivity and increasing flagellar amplitude and vigor. This type of motion is called “hyperactivation”, and it was first described in golden hamster spermatozoa [11].

The processes occurring in sperm leading to fertilization can be explored by performing in vitro studies under specific conditions in different incubation media [12]. Capacitation is supported by molecules such as albumin, a cholesterol acceptor that changes membrane fluidity, and bicarbonate, an anion that induces protein phosphorylation by stimulating adenylyl cyclase and increasing cAMP [13]. Energy substrates, such as glucose, lactate, and pyruvate, may also be needed for capacitation [14], and differences exist between species regarding the substrates required and the metabolic pathways used for energy production [15]. Extracellular calcium is also essential. Its entry into the cell during capacitation [16] relates to the modifications of the sperm membrane as well as phosphorylation in tyrosine and an increase in the pH of the acrosomal lumen [17]. Continued calcium entry produces pore formation between the outer acrosomal and plasma membranes, which accelerates acrosomal exocytosis [8]. Other biological molecules are also important in the capacitation of diverse species, including catecholamines [18,19,20], hypotaurine/taurine [21], penicillamine [22,23,24,25], a combination of penicillamine, hypotaurine, and epinephrine [26,27], melatonin [28], serotonin [29], or 5-hydroxytriptamine [30].

Progesterone is an important regulator of capacitation [31,32,33], it can trigger the acrosome reaction [34,35,36], and it changes the pattern of motility, enhancing hyperactivation at different concentrations [37,38]. Progesterone induces a dose-dependent influx of calcium before and after capacitation [39,40], and activates several intracellular signaling mechanisms [34,41]. The effects of progesterone may be mediated by three signaling pathways [39]: increase in intracellular calcium and efflux of chloride and sodium, activation of phospholipases, and phosphorylation of sperm proteins. Progesterone seems to have a non-genomic effect on spermatozoa, acting on atypical progesterone receptors present in the sperm head and the midpiece [42]. Noguchi et al. [33] proposed a regulatory mechanism in golden hamster sperm through which progesterone would induce the acrosome reaction at high concentrations by stimulating the influx of calcium and activation of phospholipase Cδ (PLCδ). At low concentrations, progesterone would activate phospholipase C (PLC), enhancing tyrosine phosphorylation and hyperactivation.

Several species of hamsters have served as models for sperm studies. Hamster spermatozoa have been described as high performers, since they show high sperm swimming velocities, with high levels of sperm ATP, probably due to intense sperm competition [27,43,44]. The golden hamster (*Mesocricetus auratus*) has been used in studies of fertilization biology [45,46,47]. Progress using this model species has helped our understanding of mechanisms of mammalian fertilization [45,48,49,50]. Chinese hamsters (*Cricetulus griseus*) have been used to examine acrosomal status in the female tract [51] and sperm–egg interactions [52,53]. Capacitation studies in this species are scarce, as optimal conditions for sperm incubation have not been obtained [27,51]. Another hamster model used in reproductive biology is the Siberian hamster (*Phodopus sungorus*), particularly because its reproductive physiology can be altered by varying the photoperiod [54]. Studies have examined in vitro and in vivo fertilization [55,56], with limited success in the improvement of in vitro fertilization rates [55]. 

To the best of our knowledge, there are very few studies on sperm performance in species of the genus *Phodopus*, and their sperm capacitation has never been addressed. In a previous study, we analyzed sperm traits in different hamster species [27]. We found higher motility, acrosome integrity, and ATP production in *Phodopus* hamsters in comparison to golden and Chinese hamsters during incubations under conditions that do not appear to support capacitation [27]. The objective of the present work was to analyze the effect of different media on changes in spermatozoa that could be compatible with capacitation and changes in kinematics related to hyperactivation in three species of *Phodopus*, i.e., *P. campbelli, P. roborovskii,* and *P. sungorus*. 

## 2. Results

### 2.1. Sperm Parameters

Spermatozoa were incubated in three different media that were based on a modified Tyrode’s solution with albumin, lactate, and pyruvate (see Section 4 for media composition). One medium did not contain bicarbonate but had Hepes (mTALP-H). The other two media had bicarbonate (mTALP-BH), and one of them also had 20 ng/mL of progesterone (mTALP-BH+P4). All media contained D-penicillamine, hypotaurine, and epinephrine (PHE). Spermatozoa were incubated in each medium for up to 4 h, under air for the first medium, and under 5% CO_2_/air for the other two media. At different times (0 h, 2 h, 3 h, and 4 h), aliquots of sperm suspension were obtained to assess sperm parameters.

**Figure 1:**
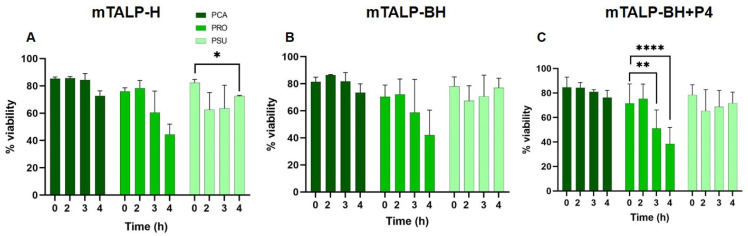
Sperm viability of three hamster species incubated in different media for 4 h. (**A**) Incubation in mTALP-H. (**B**) Incubation in mTALP-BH. (**C**) Incubation in mTALP-BH with 20 ng/mL of progesterone (mTALP-BH+P4). Abbreviations: PCA: *P. campbelli*; PRO: *P. roborovskii*; and PSU: *P. sungorus*. Asterisks indicate significant differences between times in a given species: * *p* < 0.05; ** *p* < 0.01; and **** *p* < 0.0001. Results are shown as mean ± SD.

Analysis of sperm viability (% of live cells) in mTALP-H revealed no significant changes over time in *P. campbelli* and *P. roborovskii*, whereas there was a significant decrease at 4 h in *P. sungorus* (Figure 1A). In mTALP-BH, either with or without progesterone, viability was constant over time in all species, except in *P. roborovskii*, which showed a significant decrease in viability after 3 h (*p* < 0.01) and 4 h (*p* < 0.001), when progesterone was present (Figure 1B,C). 

The comparison between spermatozoa of the different species in different media only showed differences between *P. roborovskii* (*p* < 0.05) and *P. sungorus* (*p* < 0.01) after 3 h and 4 h of incubation, when progesterone was present.

**Figure 2:**
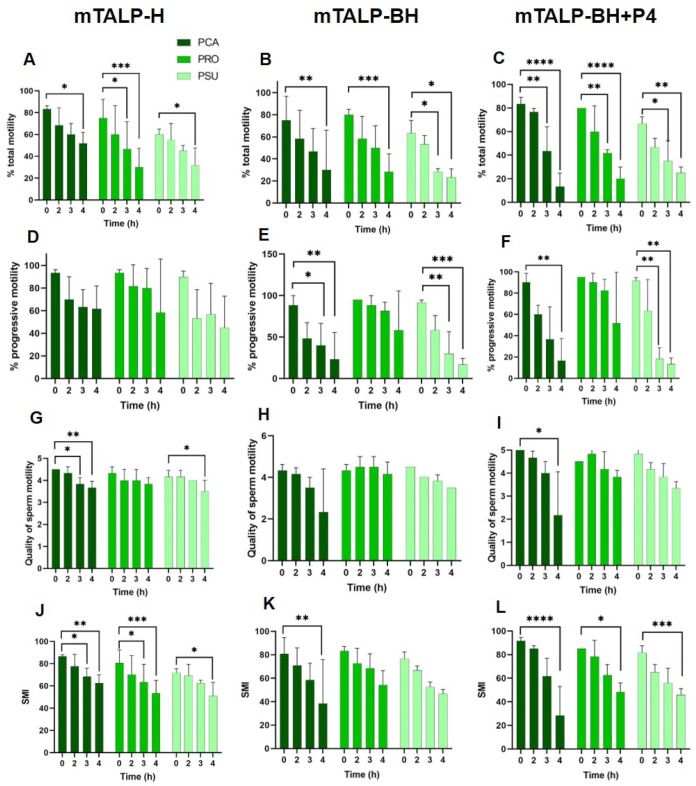
Sperm motility parameters of three hamster species incubated in different media for 4 h. (**A**–**C**) Total motility. (**D**–**F**) Progressive motility. (**G**–**I**) Quality of motility. (**J**–**L**) Sperm motility index (SMI; see Section 4). (**A**,**D**,**G**,**J**) Incubation in mTALP-H. (**B**,**E**,**H**,**K**) Incubation in mTALP-BH. (**C**,**F**,**I**,**L**) Incubation in mTALP-BH with progesterone (20 ng/mL) (mTALP-BH+P4). Abbreviations: PCA: *P. campbelli*; PRO: *P. roborovskii*; and PSU: *P. sungorus*. Asterisks indicate significant differences between species: * *p* < 0.05; ** *p* < 0.01; *** *p* < 0.001; and **** *p* < 0.0001. Results are shown as mean ± SD.

Total motility in sperm incubated in mTALP-H decreased towards the end of incubation in the three *Phodopus* species (Figure 2A), whereas the proportion of progressive sperm remained similar over time (Figure 2D). The quality of motility decreased in *P. campbelli* and *P. sungorus* at the end of incubation (Figure 2G). The sperm motility index (SMI), which is calculated based on the percentage of motility and quality (see Section 4), decreased over time in all three species (Figure 2J). Inter-species comparisons revealed no differences in total and progressive motilities, quality, or SMI at each time point assessed. 

In mTALP-BH, total motility decreased at 4 h in all species (Figure 2B). Progressive motility decreased in *P. campbelli* and *P. sungorus*, whereas it remained constant in *P. roborovskii* (Figure 2E). The quality of motility remained constant (Figure 2H) and the SMI only significantly decreased in *P. campbelli* (*p* < 0.01) (Figure 2K). The comparison of progressive motility and quality of motility between species only showed significant differences (*p* < 0.05) at 3 h of incubation (between *P. roborovskii* and *P. sungorus*) and at 4 h (between *P. campbelli* and *P. roborovskii*). The SMI of capacitated sperm was not different between species throughout time.

The addition of 20 ng/mL of progesterone (mTALP-BH+P4) resulted in a decrease in total motility at 3 h and 4 h of incubation in all species (Figure 2C). The proportion of progressive sperm was maintained in *P. roborovskii*, but it sharply decreased (*p* < 0.01) at 3 h in *P. sungorus* or 4 h in *P. roborovskii* (Figure 2F). The quality of motility was similar over time in all species, except after 4 h in *P. campbelli*, when it significantly decreased (*p* < 0.05) (Figure 2I). The SMI was reduced after 4 h in all three species (Figure 2L). 

The comparison between species revealed significantly higher total motility (*p* < 0.05) after 2 h of incubation in *P. campbelli* when compared to *P. sungorus*, as well as higher progressive motility after 3 h in *P. roborovskii* in relation to *P. sungorus*. The SMI was significantly higher after 2 h of incubation in *P. campbelli* than in *P. sungorus*.

Within species, there were differences in motility among sperm incubated in different media. The sperm of *P. campbelli* had lower values of total and progressive motility after incubation for 4 h in mTALP-BH (although marginally not significant) and in mTALP-BH+P4 (*p* < 0.05). In the other two species, there were no statistically significant differences between media at different time points.

### 2.2. Changes in CTC Staining Patterns

**Figure 3:**
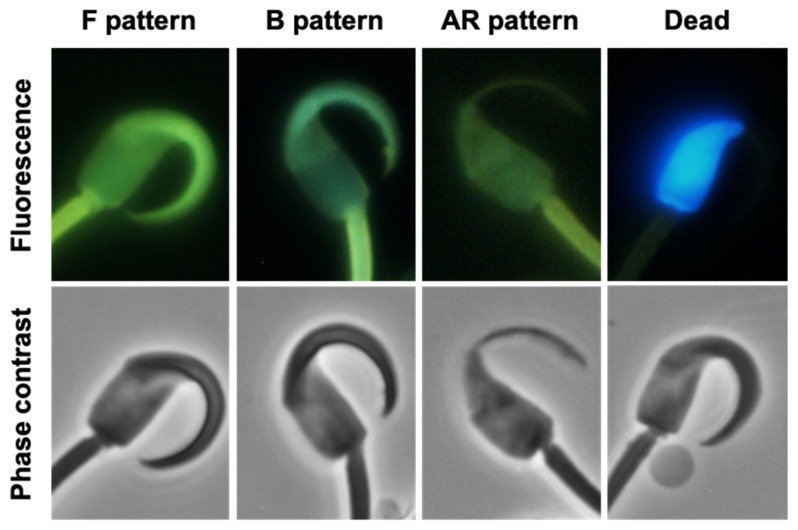
Chlortetracycline (CTC) and Hoechst 33258 staining patterns of spermatozoa from *Phodopus* species examined under fluorescence (**upper panels**) or phase contrast (**lower panels**) microscopy. The images are of *Phodopus campbelli* sperm, and the three species showed similar staining patterns. From left to right, the images correspond to **pattern “F”** live non-capacitated sperm showing a uniform bright staining of the head with CTC; **pattern “B”** live capacitated sperm showing the acrosomal area stained, contrasting with the lesser stained post-acrosomal region; **pattern “AR”** live sperm with no acrosomes and pale staining with CTC; and **dead** sperm showing a uniform staining with Hoechst 33258.

Changes in sperm staining patterns that are presumed to reflect capacitation status were evaluated after staining cells with Hoechst 33258 and chlortetracycline (CTC) (Figure 3). Live cells (i.e., those excluding Hoechst 33258) showing a uniform, bright CTC staining of the head were regarded as non-capacitated (F pattern). Live cells with acrosomes, but unstained in the post-acrosomal region, were considered to be capacitated (B pattern). Live cells with no acrosomes and pale staining were considered to have undergone the acrosome reaction (AR pattern). 

**Figure 4:**
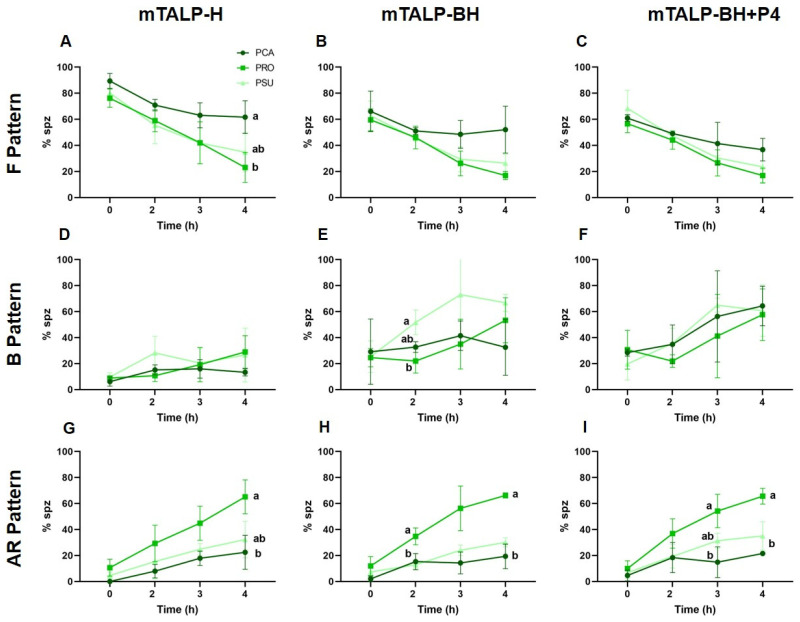
Analysis of CTC staining patterns in three hamster species incubated in different media for 4 h. (**A**–**C**) F pattern, presumably corresponding to live non-capacitated sperm. (**D**–**F**) B pattern, presumably corresponding to live capacitated sperm. (**G**–**I**) AR pattern, live sperm with no acrosome. (**A**,**D**,**G**) Incubation in mTALP-H. (**B**,**E**,**H**) Incubation in mTALP-BH. (**C**,**F**,**I**) Incubation in mTALP-BH with progesterone (20 ng/mL) (mTALP-BH+P4). Abbreviations: PCA: *P. campbelli*; PRO: *P. roborovskii*; and PSU: *P. sungorus*. Different letters at the same time point indicate significant differences between species (*p* < 0.05). Results are shown as mean ± SD.

The incubation in medium without bicarbonate (mTALP-H) appeared to result in a time-dependent decrease in F pattern cells in the three species, which was significant after 4 h in *P. roborovskii*. Comparisons between species revealed that *P. campbelli* had a significantly higher percentage of F pattern cells than *P. roborovskii* at the end of incubation (Figure 4A). The proportion of cells exhibiting a B pattern was similar over time in the three species (Figure 4D). Similarly, there were no significant increases in AR patterns over time, with the exception of *P. roborovskii*, which showed a rise after 3 h and 4 h of incubation, mirroring the observation that F pattern cells decreased in this species at the end of incubation. This rise resulted in significantly higher values of AR cells in *P. roborovskii* in comparison to values recorded in *P. campbelli* and *P. sungorus* (Figure 4G). 

In the medium with bicarbonate (TALP-BH), only *P. roborovskii* showed a significant decrease (*p* < 0.05) of F pattern cells after 3 h and 4 h of incubation. The other two species also exhibited a tendency towards a decrease in values, but, despite the differences between species, they did not reach significance (Figure 4B). Regarding B pattern cells, a significantly higher value was seen in *P. sungorus* than in *P. roborovskii* after 2 h of incubation (*p* < 0.05; Figure 4E). *P. roborovskii* showed a significant increase (*p* < 0.05) in AR pattern cells after 4 h of incubation, which was higher than that observed in the other two species from 2 h onwards (Figure 4H).

The addition of progesterone to the medium with bicarbonate (mTALP-BH+P4) led to a decrease in the proportion of cells exhibiting a F pattern; the differences observed between species did not reach significance (Figure 4C). The proportion of B pattern cells tended to increase in the three species after 2 h of incubation; however, there was considerable variation between samples and species, so statistical differences were not evident (Figure 4F). Finally, the AR pattern did not change over time in *P. campbelli*, but it increased during incubation in the other two species and was statistically significant in *P. roborovskii*; it was also significantly higher than values in the other species (Figure 4I).

Comparisons were also made between the three media used for the incubation of spermatozoa (Figure S1 and Table S1). In *P. campbelli*, there were higher percentages of F-pattern cells and lower percentages of B pattern cells over time in the medium without bicarbonate (mTALP-H) in comparison to media with bicarbonate (mTALP-BH; mTALP-BH+P4). No differences were found in cells exhibiting an AR pattern between media. In *P. roborovskii*, similar trends were observed, but their differences did not reach statistical significance. Finally, in *P. sungorus*, the same trends were found, with percentages of F pattern cells being higher in the medium without bicarbonate (although they were marginally no significant), and percentages of B pattern cells also being lower in this medium (in this case, with a significant difference). No differences were found among media in the percentage of cells with an AR pattern for each of the three species.

**Figure 5:**
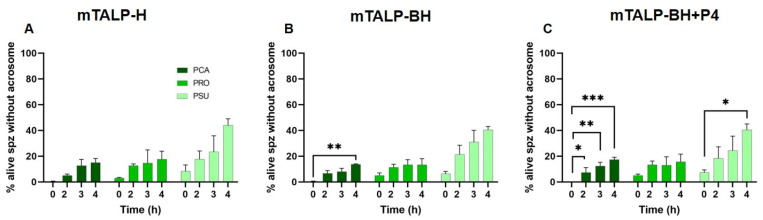
Spontaneous acrosome reactions (live sperm without acrosome) assessed using eosin–nigrosin/Giemsa in three hamster species incubated in different media for 4 h. (**A**) Incubation in mTALP-H medium; (**B**) incubation in mTALP-BH medium; and (**C**) incubation in mTALP-BH with progesterone (20 ng/mL) (mTALP-BH+P4). Abbreviations: PCA: *P. campbelli*; PRO: *P. roborovskii*; and PSU: *P. sungorus*. Asterisks indicate significant differences between times: * *p* < 0.05, ** *p* < 0.01, and *** *p* < 0.001. Results are shown as mean ± SD.

Spontaneous acrosome reactions were also assessed after staining cells with eosin–nigrosin/Giemsa stain. This staining method distinguishes live cells from dead cells and allows for the identification of acrosomal status. The results showed increases in the proportion of live cells lacking an acrosome over time in the three media with no major differences between media (Figure 5). In contrast to the results with Hoechst 33258 and CTC, *P. sungorus* was the species with higher values of acrosomal loss in live cells at the end of incubations (*p* < 0.01).

### 2.3. Sperm Kinematics

Velocity and trajectory parameters were assessed using a computer-assisted sperm analysis (CASA) system at different times of incubation (0 h, 2 h, 3 h, and 4 h) in different media (mTALP-H, mTALP-BH, or mTALP-BH+P4) in the three *Phodopus* species.

#### 2.3.1. Velocity Parameters

**Figure 6:**
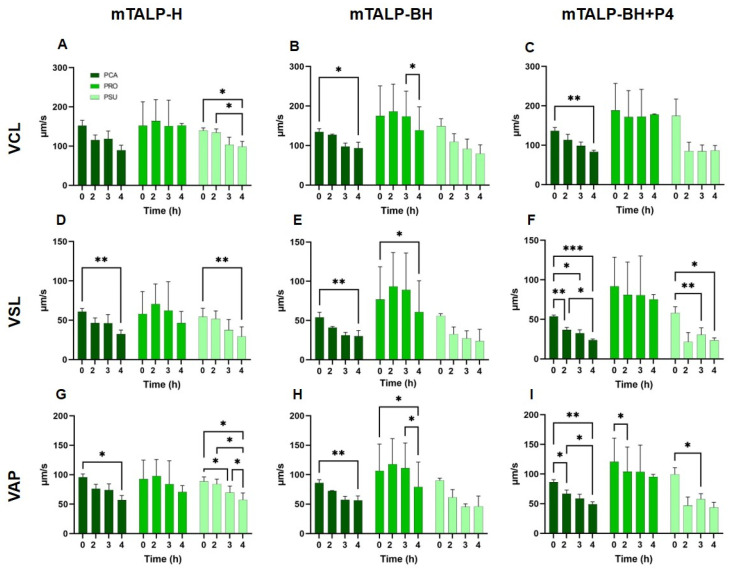
Velocity parameters of sperm kinematics in three hamster species incubated in different media for 4 h. (**A**,**D**,**G**) Incubation in mTALP-H. (**B**,**E**,**H**) Incubation in mTALP-BH. (**C**,**F**,**I**) Incubation in mTALP-BH with progesterone (20 ng/mL) (mTALP-BH+P4). Abbreviations: PCA: *P. campbelli*; PRO: *P. roborovskii*; and PSU: *P. sungorus*. Asterisks indicate significant differences between times in a given species: * *p* < 0.05; ** *p* < 0.01; and *** *p* < 0.001. Results are shown as mean ± SD.

In mTALP-H, *P. campbelli* and *P. sungorus* exhibited a significant decrease in velocity parameters towards the end of incubation. *P. roborovskii* showed no significant differences over time (Figure 6A,D,G). When comparing velocities between species, significant differences were observed in VCL between *P. roborovskii* and *P. sungorus* at times of 0 h, 3 h, and 4 h, and between *P. campbelli* and *P. roborovskii* from 3 h onwards (*p* < 0.05). VSL was significantly higher in *P. roborovskii* compared to the other two species at 3 h (*p* < 0.05), whereas VAP was not different between species. 

The incubation in mTALP-BH resulted in significant changes in velocity parameters. *P. campbelli* experienced a significant decrease (*p* < 0.05) after 4 h of incubation in all parameters, whereas *P. roborovskii* underwent a significant decline after 4 h in VSL and VAP (*p* < 0.05). In *P. sungorus*, these parameters were reduced but not significantly (Figure 6B,E,H). In general terms, *P. roborovskii* showed significantly higher velocities than the other two species. 

When sperm were incubated with progesterone in mTALP-BH (mTALP-BH+P4) for up to 4 h, their velocities experienced a reduction in *P. campbelli* (VCL, VSL, and VAP) and in *P. sungorus* (VSL and VAP) (Figure 6C,F,I). *P. roborovskii* presented higher VCL, VSL, and VAP values than the other two species. 

For each species, there were no overall differences in velocity parameters between the media.

#### 2.3.2. Trajectory Parameters

**Figure 7:**
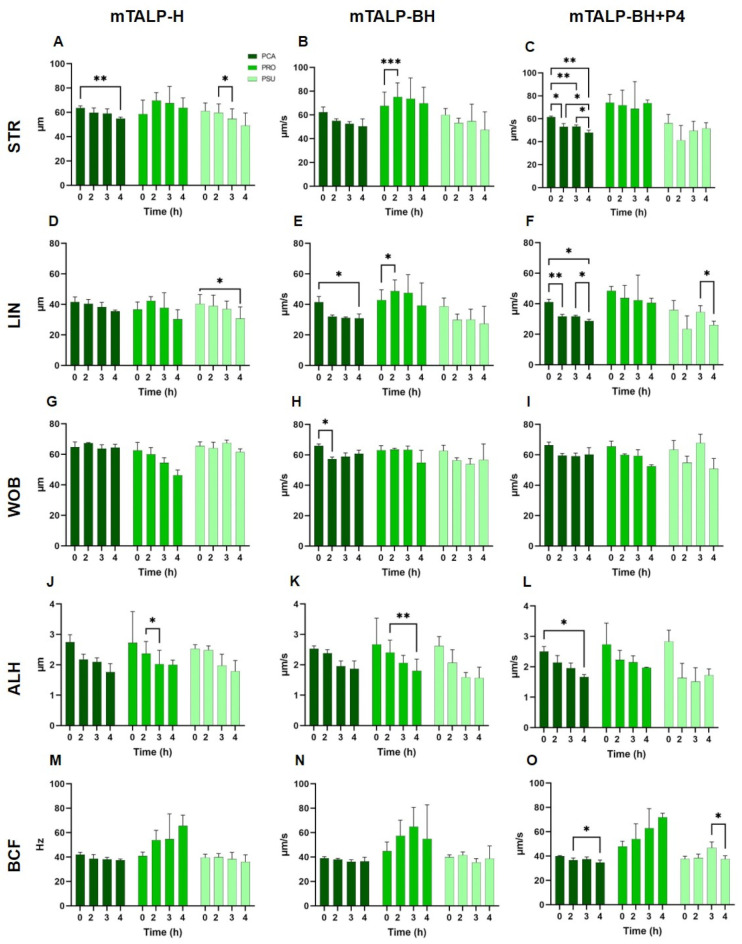
Trajectory parameters of sperm kinematics in three hamster species incubated in different media for 4 h. (**A**,**D**,**G**,**J**,**M**) Incubation in mTALP-H. (**B**,**E**,**H**,**K**,**N**) Incubation in mTALP-BH. (**C**,**F**,**I**,**L**,**O**) Incubation in mTALP-BH with progesterone (20 ng/mL) (mTALP-BH+P4). Abbreviations: PCA: *P. campbelli*; PRO: *P. roborovskii*; and PSU: *P. sungorus*. Asterisks indicate significant differences between times in a given species: * *p* < 0.05; ** *p* < 0.01; and *** *p* < 0.001. Results are shown as mean ± SD.

In mTALP-H, a significant decline was observed in STR in *P. campbelli* and in LIN in *P. sungorus* after 4 h of incubation (Figure 7). ALH significantly decreased after 2 h of incubation in *P. campbelli*. Interspecies comparisons yielded no significant differences.

In mTALP-BH, the trajectory parameters (Figure 7) of *P. campbelli* sperm showed significant decreases in LIN after 4 h of incubation (*p* < 0.05) and in WOB after 2 h of incubation (*p* < 0.05). *P. roborovskii* exhibited significant decreases in STR and LIN after 2 h of incubation (*p* < 0.01). The trajectory parameters of *P. sungorus* sperm remained unchanged over time. There were no statistical differences between species in trajectories except for WOB, which was higher in *P. roborovskii* at 2 h.

When progesterone was added to mTALP-BH (mTALP-BH+P4), *P. campbelli* sperm experienced a reduction in STR and LIN from 2 h onwards, and in ALH after 4 h (*p* < 0.05) (Figure 7). On the other hand, the trajectory values of *P. roborovskii* and *P. sungorus* remained constant over time, except for LIN in *P. sungorus*, which decreased at 4 h (*p* < 0.05). 

When comparing the three species, STR, LIN, ALH, and BCF values were higher at nearly every time point in *P. roborovskii* in relation to the other two species. 

Within species, there were some significant differences between media. In *P. campbelli* sperm, there were lower values of STR and LIN after 4 h when progesterone was present in comparison to mTALP-H. WOB was lower at 2 h in this species when incubated in mTALP-BH, with or without progesterone, in comparison to values in mTALP-H. *P. roborovskii* only showed differences between media in WOB; its value in mTALP-BH was higher than in mTALP-H at 3 h and than in mTALP-BH+P4 at 2 h. There were no differences between media in *P. sungorus*.

## 3. Discussion

In the present study, we analyzed changes in the spermatozoa of three species of *Phodopus* hamsters incubated in three different media. To the best of our knowledge, this has not been examined before in these species. The spermatozoa were incubated in modified Tyrode’s medium with albumin, lactate, pyruvate, and Hepes (mTALP-H) under air, in mTALP-H with bicarbonate under 5% CO_2_/air (m-TALP-BH), or in mTALP-BH with the addition of 20 ng/mL of progesterone (m-TALP-BH+P4), also under 5% CO_2_/air. All media contained D-penicillamine, hypotaurine, and epinephrine (PHE). It has been previously found that PHE is required to maintain motility in golden hamster spermatozoa [27,57] but that it does not improve (nor decreased) motility in *Phodopus* species [27]. Since the requirements for *Phodopus* sperm capacitation were unknown before this study, we opted to include PHE in incubation media in case they were a requirement for the acquisition of capacitation. We also explored whether progesterone had an effect on changes compatible with capacitation and hyperactivation. Progesterone is present in the oviductal fluid of the golden hamster at a concentration of 44.04–175.06 ng/mL [58]. In this species, 20 ng/mL of progesterone has been found to stimulate capacitation and hyperactivation [32,33], probably activating a tyrosine phosphorylation pathway [33].

mTALP-H medium is normally regarded as a non-capacitating medium in other species [44]. However, it was found that when *Phodopus* sperm were incubated in mTALP-H, there was a decrease in the percentage of cells exhibiting an F pattern, characteristic of non-capacitated cells. There were also decreases in the proportion of cells showing an F pattern when sperm were incubated with bicarbonate (mTALP-BH) or with bicarbonate plus 20 ng/mL of progesterone (mTALP-BH+P4), but, for the latter two media, these decreases were proportionally and significantly more pronounced. Generally, the values were lowest in *P. roborovskii* and highest in *P. campbelli*. 

The proportion of B pattern cells (regarded as capacitated sperm) remained similar among species (~20%) over time in mTALP-H. On the other hand, the proportion of cells exhibiting a B pattern experienced a significant increase when sperm were incubated in mTALP-BH, both in the absence or presence of progesterone. These results suggest that sperm from the three *Phodopus* species may be experiencing capacitation under these conditions, in agreement with what has been found before in other species when the spermatozoa have been incubated in bicarbonate-, calcium-, and albumin-containing media (e.g., mouse species [44]). In contrast to what was observed earlier in the golden hamster [32,33], or in other species (see Section 1), progesterone, at the concentration used here, did not appear to enhance the proportion of B pattern cells. This does not seem to agree with previous studies on *P. sungorus* [55], which revealed an increase in the rate of in vitro fertilization in a Tyrode’s medium with follicular fluid (which is known to contain a high concentration of progesterone). This discrepancy between our results and previous work may be due to different requirements for progesterone or to differences in progesterone-stimulated pathways.

The proportion of *Phodopus* sperm showing an AR pattern (suggesting acrosomal exocytosis) increased with time in all media (mTALP-H, mTALP-BH, and mTALP-BH+P4). The increase in AR patterns in mTALP-H was unexpected, and it could suggest loss of the acrosome due to damage or the occurrence of acrosomal exocytosis in this medium. In media with bicarbonate, there were also increases in the proportion of AR pattern cells. In these media, changes occurred in parallel to increases in the proportions of cells with a B pattern. The proportion of cells showing an AR pattern varied between species, being lowest in *P. campbelli* (~20%) and highest in *P. roborovskii* (~60%). Earlier studies in the golden hamster [59] showed that ~55% of sperm were motile and acrosome-reacted after 4 h in mTALP capacitation medium [60].

No additional increase in acrosomal loss was observed in the medium containing progesterone, and this could be due, as mentioned above, to differences with other species. Progesterone is reported to have different effects depending on its concentration. For instance, in boars, 10–100 nM of progesterone increases sperm viability, whereas in humans, 50 nM stimulates progressive motility [42]. Low concentrations of progesterone (20 ng/mL) and long capacitation times (5 h) were necessary for golden hamster sperm [32], but such concentrations were not sufficient to trigger an acrosome reaction in the golden hamster. Differences between *Phodopus* sperm and golden or Chinese hamster sperm have been previously reported [27].

Sperm motion and kinematics in *Phodopus* sperm under conditions that were presumed to be non-capacitating were previously reported [27]. In the present study, sperm of *Phodopus* species followed the same pattern of decreased motility when incubated in mTALP-H. Conversely, in mTALP-BH, with or without progesterone, there was a decline in progressive motility, which was sharper in *P. campbelli* and *P. sungorus* than in *P. roborovskii*, and which may suggest the development of hyperactivated motion. Hyperactivation is characterized by an asymmetric movement, a decrease in progressivity, an increase in flagellar amplitude, and a higher sperm vigor [11]. 

Sperm velocities, assessed with a CASA system, decreased over time in mTALP-BH in the three species. Similarly, several sperm trajectory parameters declined in the three species under these conditions. However, the expected decline in STR or LIN and concomitant increase in ALH was not evident, suggesting that hyperactivation patterns in these species may be different from the well-established patterns observed in other rodent species [44]. When comparisons between *Phodopus* species were made, *P. roborovskii* presented higher VCL, VSL, and VAP values than the other two species. Likewise, STR, LIN, ALH, and BCF values were higher at nearly every time point in *P. roborovskii* in relation to the other two species. In comparison to the golden hamster [27], the three *Phodopus* species exhibited higher values of velocity and trajectory parameters. 

Overall, the present study showed changes in *Phodopus* sperm incubated in media without or with bicarbonate, and without or with progesterone, that were consistent with the occurrence of capacitation and spontaneous acrosome reactions. Progesterone did not appear to have a marked effect on sperm function in *Phodopus*. The media used here were based on those used in previous studies in the golden hamster. The latter requires motility factors to survive and undergo capacitation, but they do not appear to be needed for *Phodopus* sperm. Therefore, additional studies will be required to further characterize capacitation and hyperactivation in *Phodopus* species. Such studies will help to understand differences in sperm function in these species with extreme phenotype and high-performing sperm cells.

## 4. Material and Methods

### 4.1. Animals

Adult males of three hamster species (*Phodopus campbelli, P. roborovskii,* and *P. sungorus*) were held in our animal facilities in individual cages and with free access to food and water. They were maintained under controlled conditions of light (14 h of light/10 h of darkness) and temperature (22–24 °C). All animal handling followed the Spanish Animal Protection Regulation RD53/2013 and the European Union Regulation 2010/63 and had the approval of CSIC’s ethics committee and the Comunidad de Madrid (28079-47-A). 

### 4.2. Animal Phenotype, Sperm Collection, and Incubation

Three animals of each species of *Phodopus* were sacrificed via cervical dislocation. Sperm were collected from the cauda epididymis by swim out, i.e., placing the cauda in the medium and puncturing them with the aid of a pair of scissors and allowing sperm cells to swim out for 10 min at 37 °C. The medium used was a Hepes-buffered modified Tyrode’s medium with albumin, lactate, and pyruvate (mTALP-H), and with D-penicillamine, hypotaurine, and epinephrine. It had the following composition: 120.89 mM NaCl, 2.68 mM KCl, 0.49 mM MgCl_2_·6H_2_O, 0.36 mM NaH_2_PO_4_·2H_2_O, 20 mM Hepes, 1.80 mM CaCl_2_, 5.56 mM glucose,1 mM sodium pyruvate, 10 mM sodium lactate, 4 mg/mL bovine serum albumin, 20 µM D-penicillamine, 100 µM hypotaurine, and 1 µM epinephrine, and had a pH = 7.4 [27].

The medium with sperm was transferred to a pre-warmed plastic tube, and the concentration of sperm was estimated using a Neubauer chamber. The samples were diluted to a final concentration of 20 × 10^6^ sperm/mL in three different media. We used mTALP-H, and a modification of this medium with 15 mM of NaHCO_3_, adjusting NaCl to 116.89 mM to maintain the osmolality (mTALP-BH). Progesterone (20 ng/mL; P0130, Sigma, Madrid, Spain) was added to the latter medium to promote sperm hyperactivation (mTALP-BH+P4). Sperm suspensions were incubated at 37 °C for 4 h under air (mTALP-H) or under 5% CO_2_/air (mTALP-BH and mTALP-BH+P4). 

### 4.3. Sperm Motility and Viability Assessment

Total and progressive motilities and quality of motility (from 1 to 5, with 1 being barely moving sperm and 5 being very vigorously moving sperm) were assessed under phase contrast at the time of dilution (time 0) and after 2 h, 3 h, and 4 h of incubation in each medium. The sperm motility index (SMI) was calculated using the equation: [((Quality of motility × 20) + Total motility)/2].

Sperm viability was measured at 0 h, 2 h, 3 h, and 4 h in each individual and each medium, examining cells after staining with eosin–nigrosin/Giemsa, as previously described [61]. Briefly, a sperm suspension was mixed 1:3 with a solution of eosin (E6003, Sigma) and nigrosin (N4754, Sigma) on a pre-warmed slide. After 30 s, smears were performed and dried at 37 °C on a warm plate. For Giemsa staining, smears were fixed with 4% formaldehyde (211328, Panreac, Madrid, Spain) for 10 min, washed, and stained for 60 min in Giemsa (109203, Merck, Madrid, Spain). The slides were mounted with DePeX (360292F, BDH, Madrid, Spain). For viability assessment, sperm with a pale post-acrosomal region were regarded as viable and those with a strong pink coloration as non-viable cells. Acrosomes were distinguished as stained with Giemsa.

### 4.4. Capacitation Analysis

Sperm aliquots were fixed 1:1 with 2% glutaraldehyde (*v*/*v*) (360802F, BDH) in 0.165 M of sodium cacodylate/HCl (301183V, BDH) at 0 h, 2 h, 3 h, and 4 h of incubation. The samples were then stained with Hoechst 33258 (B2883, Sigma) and chlortetracycline (CTC) (C4881, Sigma) to estimate the viability and the functional state (capacitation and acrosome integrity) of spermatozoa, as previously described [44]. Hoechst 33258 stains the DNA of plasma membrane-damaged cells, emitting blue fluorescence, whereas CTC stains the sites that bind calcium, staining cells with green fluorescence and showing patterns related to the cell’s capacitation and acrosomal status, namely (a) “F pattern”: non-capacitated cells (the entire sperm head is stained green with a uniform intensity); (b) “B pattern”: capacitated cells (the acrosomal region is stained green, whereas the post-acrosomal region is unstained or slightly stained); and (c) “AR pattern”: acrosome loss has occurred (sperm head slightly or not stained). Fluorescence staining was detected using a Nikon (Tokyo, Japan) UV-2A 330 nm filter for Hoechst 33258 and a Nikon BV-2A 405 nm filter for CTC. Fifty cells per sample were counted, and the percentage of each pattern was calculated. Acrosome status was also assessed in the eosin–nigrosin/Giemsa preparations.

### 4.5. Sperm Kinematics

Sperm parameters of velocity and trajectory were analyzed with a computer-assisted sperm analysis (CASA) system (Sperm Class Analyzer, SCA v.6.2, Microptic, Barcelona, Spain). Aliquots of 5 µL of a sperm suspension were diluted in 20 µL of the corresponding medium to achieve a final concentration of 4 × 10^6^ sperm/mL. Each diluted sample was loaded in a microscopy chamber with a depth of 20 μm (Leja, Nieuw-Vennep, The Netherlands), and a set of 6 videos were recorded using a phase contrast microscope, with pseudo-negative phase, connected to a digital video camera (Basler A312fc, Vision Technologies, Glen Burnie, MD, USA), as in previous studies [27].

The swimming parameters registered were the following: VCL (curvilinear velocity, μm/s), VSL (straight-line velocity, μm/s), VAP (average path velocity, μm/s), STR (STR = VSL/VAP), LIN (linearity, LIN = VSL/VCL), WOB (wobble, WOB = VAP/VCL), ALH (amplitude of lateral head displacement, μm), and BCF (beat-cross frequency, Hz).

### 4.6. Statistical Analysis

Analyses were carried out using GraphPad Prism 9 software (Dotmatics, Boston, MA, USA). Data were transformed before analyses (log_10_ for all variables, with the exception of percentages which were arcsine transformed). For the study of total and progressive motility, quality of motility, SMI, and sperm viability, two-way ANOVAs with Šídák’s multiple comparisons post-hoc tests were executed for each medium analyzed (mTALP-H, mTALP-BH, and mTALP-BH+P4), with species (*P. campbelli, P. roborovskii*, and *P. sungorus*) and time of incubation (0 h, 2 h, 3 h, and 4 h) as fixed effects, including their interaction in the statistical model. Capacitation patterns were assessed using a two-way ANOVA with Tukey’s post-hoc test, considering species and time as fixed effects. Finally, differences in sperm kinetics between species at the same time point were analyzed using a two-way ANOVA with Tukey’s post-hoc test, whereas the differences between times in the same species were examined using a one-way ANOVA. All variables are shown as means ± SD, and significance is marked with asterisks depending on the level of significance (* *p* < 0.05, ** *p* < 0.01, *** *p* < 0.001, and **** *p* < 0.0001).

## Data Availability

The raw data supporting the conclusions of this article will be made available by the authors, without undue reservation.

## Supplementary Materials

The supporting information can be downloaded at: https://www.mdpi.com/article/10.3390/ijms242216093/s1.
