# Supplementary material for: Sperm Capacitation and Kinematics in Phodopus Hamsters
Source: Int J Mol Sci. 2023 Nov 8;24(22):16093. doi: 10.3390/ijms242216093 (PMC10671044; doi:10.3390/ijms242216093)
Supplement: Supplementary file 1 [file ijms-24-16093-s001.zip › ijms-2654457-supplementary.pdf]

Supplementary Material

Sperm Capacitation and Kinematics in *Phodopus* hamsters

Ana Sanchez-Rodriguez, Ingrid I. D. Idrovo, Juan Antonio Rielo and Eduardo R. S. Roldan

Department of Biodiversity and Evolutionary Biology, Museo Nacional de Ciencias Naturales (CSIC), calle Jose Gutierrez Abascal 2, 28006-Madrid, Spain

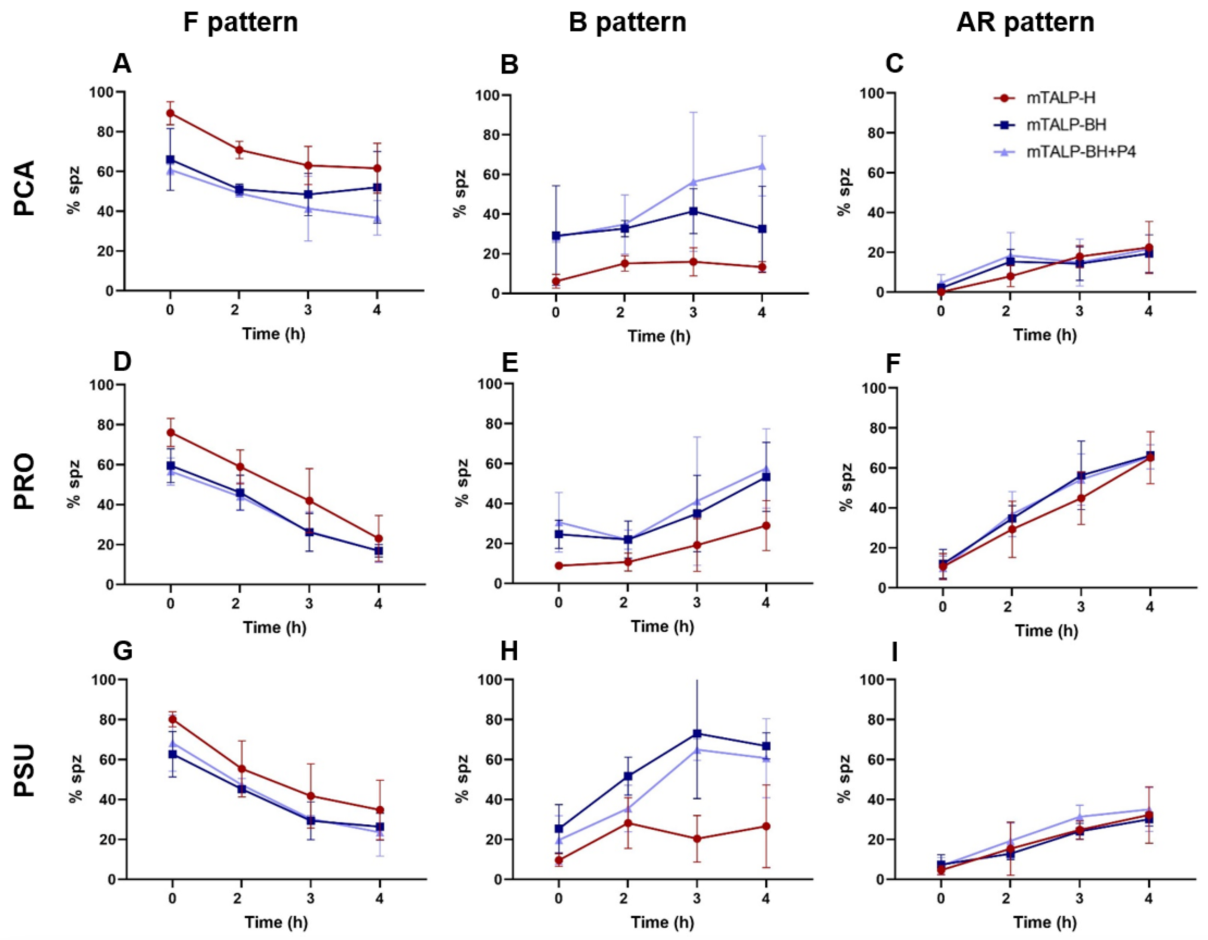

Figure S1. Comparisons between media used to incubate spermatozoa of three species of *Phodopus*. Spermatozoa were incubated for 4 h in a modified Tyrode medium with albumin, lactate, and pyruvate and with Hepes but without bicarbonate (mTALP-H), the same medium also containing bicarbonate (mTALP-BH) or medium containing bicarbonate and 20 ng of progesterone/ml (mTALP-BH+P4). At different times spermatozoa were sampled and stained with CTC. Percentages of cells showing an F pattern (**A,D,G**), B pattern (**B,E,H**) or AR pattern (**C,F,I**) were quantified. Abbreviations: PCA, *P. campbelli*; PRO, *P. roborovskii*; and PSU, *P. sungorus*. Results are means ± SD. For statistics see Table S1.

Table S1. Results of 2-factor ANOVAs for the comparisons between media used to incubate spermatozoa of three species of *Phodopus*. Spermatozoa were incubated for 4 h in a modified Tyrode medium with albumin, lactate, and pyruvate, and with Hepes but without bicarbonate (mTALP-H), the same medium also containing bicarbonate (mTALP-BH) or medium containing bicarbonate and 20 ng of progesterone/ml (mTALP-BH+P4). At different times spermatozoa were sampled and stained with CTC, and percentages of F pattern, B pattern and AR pattern cells were quantified. In bold, statistically significant values.

Species	CTC pattern	Fixed effects (type III)	*P* value	*F*
P. cambelli	F	Time	**0.0003**	12.64
		Media	**0.0252**	7.239
		Time x Media	0.8132	0.4812
	B	Time	0.1605	2.213
		Media	**0.0078**	12.12
		Time x Media	0.5881	0.7929
	AR	Time	**0.0016**	11.35
		Media	0.8568	0.1586
		Time x Media	0.6309	0.7316
P. roborovskii	F	Time	**< 0.0001**	88.56
		Media	0.1529	2.61
		Time x Media	0.5854	0.7968
	B	Time	**0.0079**	9.356
		Media	0.1533	2.605
		Time x Media	0.9205	0.3146
	AR	Time	**< 0.0001**	75.59
		Media	0.8188	0.2068
		Time x Media	0.8857	0.3736
P. sungorus	F	Time	**< 0.0001**	32.87
		Media	0.0913	3.662
		Time x Media	0.9846	0.1584
	B	Time	**0.0072**	12.24
		Media	**0.0087**	11.60
		Time x Media	0.1742	1.765
	AR	Time	**< 0.0001**	24.94
		Media	0.4962	0.7893
		Time x Media	0.9631	0.2256
